# Reemergence of chloroquine-sensitive *pfcrt* K76 *Plasmodium falciparum* genotype in southeastern Cameroon

**DOI:** 10.1186/s12936-017-1783-2

**Published:** 2017-03-27

**Authors:** Nicaise Tuikue Ndam, Leonardo K. Basco, Vincent Foumane Ngane, Ahidjo Ayouba, Eitel Mpoudi Ngolle, Philippe Deloron, Martine Peeters, Rachida Tahar

**Affiliations:** 1 UMR 216 Mère et Enfant Face aux Infections Tropicales, Institut de Recherche pour le Développement (IRD), 4, Avenue de l’Observatoire, 75270 Paris, France; 2 PRES Sorbonne Paris Cité, Université Paris Descartes, Faculté de Pharmacie, 75270 Paris, France; 3 Noguchi Memorial Institute for Medical Research, University of Ghana, P.O. Box LG 581, Accra, Ghana; 4 Unité de Recherche sur les Maladies Infectieuses et Tropicales Emergentes (URMITE), CNRS 7278, IRD 198, INSERM 1095, AP-HM, Aix-Marseille Université, Institut Hospitalo-Universitaire (IHU)-Méditerranée Infection, 13385 Marseille, France; 5 Laboratoire de Recherche sur le Paludisme, Organisation de Coordination pour la lutte contre les Endémies en Afrique Centrale (OCEAC), B. P. 288, Yaoundé, Cameroon; 6 Unité IRD 233 Recherches Translationnelles sur le VIH et les maladies infectieuses, Montpellier, France; 7 Prévention du Sida au Cameroun, Institut de Recherches Médicales et d’Études des Plantes Médicinales, Yaoundé, Cameroon

**Keywords:** Drug resistance, Chloroquine, Artemisinin, Molecular markers, Cameroon

## Abstract

**Background:**

Chloroquine had been used extensively during the last five decades in Cameroon. Its decreasing clinical effectiveness, supported by high proportions of clinical isolates carrying the mutant *pfcrt* haplotype (CVIET), led the health authorities to resort to amodiaquine monotherapy in 2002 and artemisinin-based combination therapy (ACT) in 2004 (artesunate–amodiaquine, with artemether–lumefantrine as an alternative since 2006) as the first-line treatment of uncomplicated malaria. The aim of the present study was to investigate whether the withdrawal of chloroquine was associated with a reduction in *pfcrt* mutant parasite population and reemergence of chloroquine-sensitive parasites in southeastern Cameroon between 2003 and 2012.

**Methods:**

The frequency of *pfcrt* haplotypes at positions 72–76 in *Plasmodium falciparum* isolates collected from individuals in 2003 and 2012 in southeastern Cameroon was determined by sequence specific oligonucleotide probes-enzyme linked immunosorbent assay (SSOP-ELISA).

**Results:**

The proportions of parasites carrying the mutant haplotype CVIET and the wild-type CVMNK were 53.0 and 28.0% in 2003, respectively. The proportion of the mutant haplotype in samples collected 9 years later decreased to 25.3% whereas the proportion of parasites carrying the wild-type CVMNK haplotype was 53.7%.

**Conclusions:**

Even though the proportion of chloroquine-sensitive parasites seems to be increasing in southeastern Cameroon, a reintroduction of chloroquine cannot be recommended at present in Cameroon. The current national anti-malarial drug policy should be implemented and reinforced to combat drug-resistant malaria.

## Background

Chloroquine resistance (CQR) was first reported almost simultaneously at the Thai–Cambodian border and in the Amazon basin in the late 1950s [1, 2]. It spread from these two foci to other regions in South America, Southeast Asia, and India during the 1960s and 1970s, and to East Africa in the late 1970s. Almost all sub-Saharan African countries reported chloroquine-resistant *Plasmodium falciparum* in the 1980s and 1990s. In Cameroon, the first chloroquine-resistant cases were described after a chemoprophylactic failure in non-immune visitors in 1985 [3].

Chloroquine and amodiaquine were the first-line drugs for the treatment of uncomplicated malaria until 2002 in Cameroon. A series of clinical studies using the standardized World Health Organization (WHO) protocol to assess anti-malarial drug efficacy had shown the general inefficacy of chloroquine throughout the country, leading to the gradual withdrawal of chloroquine and the use of amodiaquine as the first-line drug for the treatment of uncomplicated malaria during the transition period between 2002 and 2004. This was followed by the adoption of artemisinin-based combination therapy (ACT) in 2004 (artesunate–amodiaquine, with artemether–lumefantrine as an alternative ACT since 2006) as first-line treatment [4–6]. These studies showed that chloroquine was ineffective in central, southern, eastern, and western Cameroon, but had lower treatment failure rates in the northern Sahelian region. Sulfadoxine–pyrimethamine had been the second-line drug until 2004 and, since then, has been employed for intermittent preventive treatment in pregnant women. The importation of chloroquine has been suspended since 2002 and the drug has been withdrawn from the official outlets by Cameroonian health authorities. Although chloroquine may still be available in the country through illicit trade, it can be presumed that official government measures have contributed to the reduction of drug pressure on the parasites due to chloroquine during the last decade.

*Plasmodium falciparum* chloroquine resistant transporter (*pfcrt*) was identified as a CQR marker by genetic cross between a chloroquine-resistant clone (Dd2/Indochina) and a chloroquine-sensitive clone (HB3/Honduras). A single nucleotide polymorphism (SNP) encoding an amino acid change at codon 76 (K76T) is highly correlated with both in vitro CQR and chloroquine treatment failure [7]. Additional mutations in codons 72–75 have been suggested to play a modulator role in resistance or to compensate the deleterious effect of K76T [8]. This gene is highly polymorphic and contains eight point mutations M74I, N75E, K76T, A220S, Q271E, N326S, I356T, R371I found in the Asian and African CQR isolates and in the CQR Dd2 strain [8]. The I356T mutation is absent in some resistant isolates. All these mutations localize within or near the ten predicted transmembrane segments and seem to be involved in hydrophilicity [8, 9]. Other novel mutations that have been reported include H97L/K, A144F/Y, L148I, L160Y, I194T, T333/S, S334N, C350R [9–15]. Recently, it was demonstrated that the full activity of PFCRT is a rigid process that requires additional mutations occurring in a specific order to prevent the reduction of chloroquine transport, and that a minimum of two mutations are sufficient to decrease chloroquine transport while four mutations confer full activity [16]. At present, the exact process leading to chloroquine resistant phenotype is not fully elucidated and different laboratory models suggest a multigenic basis of resistance. In this study the dynamics of the prevalence of the major *pfcrt* haplotypes between 2003 and 2012 were investigated.

## Methods

Fingerprick capillary blood samples were collected on Isocode Stix filter papers (Schleicher and Schuell, Ecquevilly, France) in the clinical study carried out in July–August 2003 in Bertoua, Cameroon. This was part of a randomized study that compared the efficacy of amodiaquine, sulfadoxine–pyrimethamine, and amodiaquine–sulfadoxine–pyrimethamine combination in children aged less than 5 years old with uncomplicated malaria [5, 6]. In 2003, chloroquine (both good quality and poor quality medicines, including counterfeit drugs) was widely available throughout the country, and this drug had been used massively for self-medication [17]. Additional venous blood samples were collected into ethylenediaminetetraacetic acid (EDTA)-coated tubes from asymptomatic individuals during a human immunodeficiency virus (HIV) survey conducted in Messok in October 2012.

Previous molecular studies in Cameroon have shown that asymptomatic parasite carriers and symptomatic patients are infected with similar proportions of dihydrofolate reductase (*dhfr*) mutant *P. falciparum* isolates in a given study site [18].

Bertoua and Messok are located in the eastern province in southeastern Cameroon. Bertoua is the regional capital of the largest forest area of the country, including the district of Messok which occupies an area of 14,500 hectares covered by forest between Dja and Boumba Bek natural reservations. Bertoua is a semi-urban area with a population of 218,111, and Messok is a rural area where most of the population of 6412 inhabitants practice hunting for living. The Guinea-type equatorial climate in Bertoua and Messok is characterized by the alternation between two rainy and two dry seasons. Mean annual rainfall varies from 1500 to 2000 mm in Bertoua and 1600 to 1700 mm in Messok. The mean annual temperature in Bertoua and Messok fluctuates between 23 and 25 °C.

Genomic DNA was extracted directly from blood using QIAamp^®^ DNA extraction kit (Qiagen, Courtaboeuf, France) according to the manufacturer’s instructions, or from filter papers using the boiling method [19]. A fragment of *pfcrt* spanning codons 72–76 was amplified by nested PCR, as described by Djimdé et al. using the following primers: TCRP-1 (5′-CCGTTAATAATAAATACACGCAG-3′) and TCRP-2 (5′-CGGATGTTACAAAACTATAGTTACC-3′) for the primary PCR and TCRD-1 (5′-TGTGCTCATGTGTTTAAACTT-3′) and TCRD-2 (5′-CAAAACTATAGTTACCAATTTTG-3′) [7]. The polymorphism at positions 72–76 was determined using sequence specific oligonucleotide probes-enzyme linked immunosorbent assay (SSOP-ELISA) based technique in which SNPs are visualized in PCR-based ELISA as described by Alifrangis et al. [20]. Proportions were compared by the Chi square test. The significance level was fixed at 0.05.

## Ethics

Research performed here was in accordance with the Declaration of Helsinki. The protocol, informed consent documents, relevant supporting information, and all patient recruitment information were approved by Cameroonian national ethics committee, Cameroonian Ministry of Public Health, and the Ethics committee of Institut de Recherche pour le Développement (France). Written informed consent was given by all participants.

## Results

A total of 179 febrile children aged less than 5 years old were recruited in the clinical study in Bertoua in 2003. The mean age (±standard deviation) was 29 ± 17 months (range 6–58 months old). The sex ratio was 0.97 (88 boys and 91 girls). The minimum required parasitaemia for inclusion was 2000 asexual parasites/µl of blood. Parasitaemia ranged from 2000 to 250,000 asexual parasites/µl of blood. The clinical and parasitological outcome was published elsewhere [5, 6].

Among 101 participants of the survey conducted in Messok in 2012, data on age and gender were available for 96 and 95 individuals, respectively. The median age at inclusion was 36 years (interquartile range, 25–55 years old). There were 38 men and 57 women. Microscopic examination of blood smears for malaria detection was not performed. Individuals of all ages were included in the study. PCR showed that almost all included individuals (100 of 101, 99%) were asymptomatic carriers of *P. falciparum*. They were not treated with anti-malarial drugs.

The genotype of 178 isolates from Bertoua and 100 isolates from Messok was analysed at amino acid positions 72–76. Of 178 and 100 samples from Bertoua and Messok, amplified fragments were successfully obtained in 164 (92%) and 95 (95%) samples, respectively. The results are summarized in Table 1. All PCR-positive samples were examined by SSOP-ELlSA assay. Among 164 isolates collected in Bertoua in 2003, 87 (53%) carried the mutant CVIET haplotype, 46 (28%) carried the wild-type CVMNK haplotype, and 31 (19%) had mixed alleles. In sharp contrast, among 95 samples collected in 2012 in Messok, only 24 (25.3%) carried the mutant CVIET haplotype, 51 (53.7%) had the wild-type CVMNK haplotype, and 20 (21.1%) were mixed. The alternative South American-type mutant haplotype SVMNT was not detected.

**Table 1:** Prevalence of *pfcrt* haplotypes in Bertoua and Messok, southeastern Cameroon

Haplotype^a^	Bertoua 2003 (n = 164)		Messok 2012 (n = 95)	
	n	%	n	%
CVMNK	46	28.0	51	53.7
CVIET	87	53.0	24	25.3
Mixed	31	18.9	20	21.1

*n* number of isolates

^a^CVMNK (wild-type) and CVIET (mutant) haplotypes indicate amino acids at positions 72–76 in PfCRT. The South American-type SVMNT haplotype was not observed in the present study. PCR failed in 14 and 5 samples from Bertoua and Messok, respectively

## Discussion

The results observed in Bertoua in 2003 are consistent with the high rate of chloroquine treatment failures observed in this region between 1999 and 2001, which led to the suspension of chloroquine use in the country [5]. The proportion of isolates with mutant *pfcrt* haplotype in Bertoua was slightly lower than that observed in isolates collected in Yaoundé during the same study period. A study conducted in Yaoundé in 2000–2001 showed a high correlation between in vitro response and *pfcrt* haplotype, indicating the usefulness of *pfcrt* to monitor CQR [21]. The similar proportions of *P. falciparum* isolates carrying the mutant *pfcrt* haplotype in Bertoua in 2003 (72%, including mixed alleles) and Yaoundé in 2000–2001 (70%, including mixed alleles) were most likely related to the widespread occurrence of chloroquine-resistant *P. falciparum* and intensive use of chloroquine, mainly for self-medication, during the pre-ACT period when chloroquine was still widely available in Cameroon. This observation is supported by another study conducted in Yaoundé in 2004–2006 which showed that 77% of clinical isolates carried the mutant 76T allele [22].

Although Messok is situated in the same province as Bertoua, there are no previous clinical or molecular data on *P. falciparum* malaria in this village. The molecular results of the present study showed an inverse relationship between the samples collected earlier in Bertoua and those collected at a later date in Messok (*P* < 0.05). This inverse relationship is in agreement with high rate of chloroquine treatment failure observed between 1999 and 2004 in nine sentinel sites of southern Cameroon [5]. The proportion of wild-type *pfcrt* almost doubled and that of mutant *pfcrt* halved after an interval of 9 years in the southeastern province of Cameroon. During this lapse of time, it can be safely assumed that drug pressure due to chloroquine declined with the suspension of drug importation into the country, as well as in other neighboring countries in Central Africa. Moreover, despite the availability of chloroquine of doubtful quality through informal drug outlets until recent years, retrospective analysis of clinical records of malaria-infected patients enrolled in clinical studies between 2005 and 2009 in Yaoundé suggests that self-medication with chloroquine has decreased considerably, as supported by negative Saker–Solomon’s urine test for anti-malarial drugs (unpublished data, Basco, personnel communication).

The reintroduction of *P. falciparum* isolates carrying wild-type *pfcrt* allele was first described in Malawi, and subsequently in other African countries as a consequence of the absence of drug pressure due to chloroquine [23–25]. The results of the present study are in agreement with the earlier studies. However, the switch from chloroquine to amodiaquine alone and then to artesunate–amodiaquine as the first-line treatment for uncomplicated malaria in Cameroon suggests that amodiaquine may not exert the same pressure as chloroquine to select K76T *pfcrt* mutant genotypes.

It is generally believed that residual chloroquine-sensitive *P. falciparum* population expands in a predominantly chloroquine-resistant zone when drug pressure is relieved and outgrows the drug-resistant population in areas of intense transmission. Alternatively, in some cases, *P. falciparum* may have a high genetic plasticity allowing this parasite to recover its primordial state of chloroquine-sensitive phenotype. In French Guiana, C350R amino acid change in *pfcrt* mutant isolates carrying K76T substitution resulted in chloroquine-sensitive phenotype [24]. Although it is still unknown if other genetic changes elsewhere in the genome could occur during this process, and to what extent these changes can influence the transport function of PfCRT and other related proteins, available data suggest that reversion of CQR in Africa is largely due to the replacement of *pfcrt* mutant isolates by wild-type isolates [26]. A genome-wide monitoring of African isolates is required to follow future evolution of parasite populations and find the potential genetic changes that may possibly occur in wild-type and mutant *pfcrt* parasites.

In the present study, a significant increase in the proportion of parasites carrying the wild-type *pfcrt* K76 haplotype was observed in Messok, but the proportion of mutant and mixed alleles was still relatively high (25 and 21%, respectively), possibly suggesting a slow process of regaining chloroquine-sensitive genotype at the parasite population level in areas of intense transmission. This observation is also compatible with a limited but continuous unauthorized use of chloroquine in the southeastern province. Other still unidentified local factors may be involved in maintaining a high proportion of *pfcrt* mutants. In a study that analysed *pfcrt* polymorphisms in clinical isolates in Yaoundé during the early years of ACT era (2005–2009), 71% of the isolates (81%, including mixed alleles) still carried the mutant 76T allele [27]. Although the number of isolates from each study site was limited (n = 24 from Yaoundé and n = 21 from Bertoua), a general trend towards a slightly lower prevalence (56%, 100 of 180 isolates) of mutant *pfcrt* CVIET haplotype was shown in five cities located in southern Cameroon in 2012, and rare mutant haplotypes (SVMNT, SVMET, CVMDT, CVMET, CVMNT) were found in 8% (15 of 180) of isolates [28, 29].

It may be hypothesized that the current nationwide use of artesunate–amodiaquine may be contributing to the maintenance of mutant *pfcrt* haplotype. Selection of parasites with K76T substitution occurs with amodiaquine monotherapy failure [30]. However, there is some contradictory evidence that similar selection of *pfcrt* mutants may not occur after artesunate–amodiaquine failure [31]. Further molecular analysis of recrudescent parasites after ACTs will be required to characterize the genotypes of recrudescent parasites.

Since more than a decade ago, chloroquine had been gradually withdrawn from the official outlets in Cameroon and elsewhere in Africa. As a consequence, many countries have reported a decreasing trend of prevalence of *P. falciparum* isolates carrying K76T mutant *pfcrt* allele [23–25]. Although the present study is limited by the comparison of data from two different sites within the same province in Cameroon and relatively small sample size, the results are consistent with decreased drug pressure due to chloroquine and in agreement with similar changes reported from other African countries.

## Conclusions

If the proportion of chloroquine-resistant parasites declines in Africa to an undetectable level of *pfcrt* mutants, a reintroduction of chloroquine in combination with other anti-malarial for treatment and prophylaxis may possibly be considered. However, at present, it is more important to ensure the rational use of highly effective ACTs, together with various chemopreventive strategies (e.g., intermittent preventive treatment in pregnancy and in infants, seasonal malaria chemoprevention) and insecticide-impregnated bed nets, to control *P. falciparum* malaria. Further studies should be conducted to prevent the emergence of resistance to other drugs.

## Abbreviations

ACT: artemisinin-based combination therapy
SSOP-ELISA: sequence specific oligonucleotide probes-enzyme linked immunosorbent assay
WHO: World Health Organization
*pfcrt*: *P. falciparum* chloroquine resistant transporter
CQR: chloroquine resistant
